# The PRISMA 2020 statement in Portuguese: updated recommendations for reporting systematic reviews

**DOI:** 10.1590/SS2237-9622202200011

**Published:** 2022-07-22

**Authors:** Taís Freire Galvão, Gustavo Magno Baldin Tiguman, Rafael Sarkis-Onofre

**Affiliations:** 1 Universidade Estadual de Campinas, Faculdade de Ciências Farmacêuticas, Campinas, SP, Brazil; 2 Faculdade Meridional, Escola de Saúde, Passo Fundo, RS, Brazil

After more than a decade since the preparation and publication of the Preferred Reporting Items for Systematic Reviews and Meta-Analyses (PRISMA) statement in 2009, the PRISMA 2020 statement provides updated recommendations that reflect advances in methods in this area.^1^ A review of 60 documents led to the identification of items in the PRISMA 2009 statement that needed to be changed. The changes were analyzed by 110 researchers and the approved version was evaluated in a face-to-face meeting to review the wording of the items and other enhancements to ensure the clearness of the guideline.^2^

The PRISMA statement aims to ensure transparent reporting of systematic reviews, their methods and findings. The PRISMA 2020 guideline defines the minimum set of evidence-based items for reporting systematic reviews and meta-analyses, as follows: i) a checklist with 27 items; ii) an expanded checklist; iii) a checklist for abstracts; and iv) flow diagrams for both new and updated systematic reviews.^1^ The statement is mainly intended for systematic reviews that evaluate the effects of interventions, although it can be used as guidance for reporting in systematic reviews involving observational studies, such as prevalence studies. For authors of systematic reviews and meta-analyses, the statement contributes directly to the drafting of their manuscripts. It also helps in the assessment of systematic reviews intended for publication by informing the minimum set of items that a given manuscript should report, thus supporting peer reviewing and editorial appraisal. Endorsement of the PRISMA statement by journals, with its inclusion in their instructions for authors, is encouraged by the group that prepared the updated statement, and contributes to its ultimate objectives of improving systematic review reporting.^1^

It should be emphasized that the PRISMA instrument is not intended for critical appraisal of systematic reviews. Neither is it a guide for conducting systematic reviews with regard to methodological procedures to be used in this type of research. Transparency and reproducibility of systematic reviews are enhanced when they comply with the PRISMA statement. These reporting characteristics do not ensure use of the best research methods, which presupposes rigor in the processes of preparing the research question and eligibility criteria, selection, extraction and critical appraisal of the articles included, synthesis and assessment of the certainty of the new evidence.^3^ ^-^ ^7^ The measurement tool for Assessment of Multiple Systematic Reviews (AMSTAR) was developed for this purpose,^8^ and its updated version (AMSTAR-2) is recommended for assessing the methods used in systematic reviews.

On the one hand, endorsement of the statement by journals does not, by itself, ensure improved systematic review reporting quality,^11^ and strategies to improve adherence to complete and transparent reporting in this form of research need to the tested and put into place. On the other hand, full reporting of the procedures employed and results found in systematic reviews is assumed to increase the quality of such research indirectly. Adherence to the PRISMA 2009 checklist and compliance with the AMSTAR instrument were significantly greater in journals in the field of gastroenterology and hepatology that endorsed PRISMA.^9^ Systematic reviews that followed the PRISMA 2009 guidelines had better reporting quality in reviews about nursing interventions in individuals with Alzheimer's disease, and reviews with meta-analyses and a registered protocol (PRISMA items) had greater methodological quality as assessed by AMSTAR.^10^

In the Brazilian context, adopting PRISMA 2020 will potentially contribute to the better dissemination of evidence found by systematic reviews in the Portuguese language and may therefore contribute to building more solid guidelines within the Brazilian National Health System (*Sistema Único de Saúde* - SUS).

Epidemiology and Health Services: Journal of the Brazilian National Health System (*Epidemiologia e Serviços de Saúde: revista do SUS* - RESS) has endorsed the PRISMA statement right from its first version and its instructions for authors include the recommendation to follow this reporting guideline when submitting systematic reviews. In 2015, RESS published the Portuguese version of the statement, after having undertaken its translation and back-translation, which were then validated by the group that prepared the statement.^12^ At the time of the translation of the PRISMA 2009 statement to Portuguese, a series of methodological articles explaining the process of carrying out systematic reviews was organized and published by RESS,^3^ ^-^ ^8^ so as to support researchers in conducting this form of research. As part of the effort to make the 2020 version of the PRISMA statement available in Portuguese, it was translated and back-translated and submitted for appraisal by the group that prepared the 2020 version. That group approved the Portuguese version of the PRISMA 2020 statement, which has now been published on the RESS website along with the checklists that are available for downloading.^13^

RESS reaffirms its commitment to the quality of research and its reporting and contributes to the dissemination of the PRISMA 2020 statement for writing systematic reviews in Portuguese. Such research supports and informs the incorporation of technologies and other recommendations based on scientific evidence in the SUS. 
